# Epidemiologic evaluation of head and neck patients in a university hospital of Northwestern São Paulo State

**DOI:** 10.1016/S1808-8694(15)30753-9

**Published:** 2015-10-19

**Authors:** Larissa de Melo Alvarenga, Mariangela Torreglosa Ruiz, Érika Cristina Pavarino-Bertelli, Maurício José Cabral Ruback, José Victor Maniglia, Eny Maria Goloni-Bertollo

**Affiliations:** 1 Undergraduate Nurse; 2 Master's degree in Biological Science, Biologist – Sao Jose do Rio Preto Medical School; 3 Doctorate in Biological Science, Adjunct Professor of Genetics – Sao Jose do Rio Preto Medical School; 4 Physician, Otorhinolaryngology and Head & Neck Surgery Specialist; 5 Associate Professor (livre-docente habilitation) of Otorhinolaryngology and Head & Neck Surgery; 6 Associate Professor (livre-docente habilitation) of Human and Medical Genetics, Associate Professor (livre-docente) of the Genetics Discipline, Sao Jose do Rio Preto Medical School

**Keywords:** epidemiology, and neck neoplasia, tobacco and alcohol

## Abstract

Head and neck cancer accounts for nearly 200.000 new cases worldwide. A mean of 13.470 new cases of cancer in the oral cavity for 100.000 inhabitants is observed in Brazil.

**Aim:**

To analyze clinical and epidemiological aspects in patients consulted in the Otorhinolaryngology and Head and Neck Surgery ward in a University hospital of Northwestern São Paulo, Brazil.

**Materials and Methods:**

A total of 427 patients consulted in the hospital in the period from 2000 to 2005 were investigated. The variables analyzed included: age, gender, occupation, skin color, tobacco and alcohol consumption, primary site of the tumor, clinical staging, degree of histological differentiation and outcome. The data was analyzed by descriptive and exploratory statistics.

**Results:**

Prevalence was found among men (86%), white color (90%), smokers (83.37%), and alcoholics (65.80%); the average age was 61 years, 24.25% of men were farmers and 60% of women, housekeepers. Primary site of tumor was usually in the oral cavity (35.37%), with histological squamous cell. The incidence of deaths was 164.

**Conclusion:**

This study has provided the profile of the patients assisted in this hospital; moreover, it has contributed to outline further programs for preventing this disease.

## INTRODUCTION

Head and neck cancer is a collective term based on anatomical and topographic definitions for describing malignant tumors of the upper aerodigestive tract. This anatomical region comprises the oral cavity, the pharynx and the larynx. “Oral cancer” is one of the major subgroups of head and neck carcinomas; it involves the mucosa of the mouth (lips, base of tongue, tongue, floor of the mouth and the hard palate) and pharynx (oropharynx, hypopharynx and nasopharynx). About 40% of head and neck cancers occur in the oral cavity, 15% occur in the pharynx, 25% occur in the larynx and the remaining tumors occur in other sites (salivary glands and thyroid)^1^. The most frequent histological type, occurring in over 90% of cases, is the squamous cell carcinoma.^2^

This disease is responsible for many deaths worldwide; it is the sixth cause of death by cancer. Each year approximately 200 thousand new cases of head and neck cancer are diagnosed worldwide.^3^ In Brazil, estimates indicate that there will be approximately 13,470 new cases of oral cavity cancer for each 100 thousand persons in 2008 (10,060 estimated in males and 3,410 estimated cases in females).4 The incidence of mouth cancer in Brazil is 2% of all cancers, one of the highest in the world, and significant in Latin America.^5^ The estimated mortality rate is approximately 12,300 deaths per year;^6^ the survival rate is 40 to 50% for diagnosed patients.^7^^,^^8^

Epidemiological evidence shows that the incidence of head and neck cancer increases with age. In Europe, 98% of the patients are aged over 40 years.^1^ This type of tumor is rare in young patients, involving only 4 to 6% of persons aged below 40 years, although this incidence has increased in a number of countries,^9^ The carcinogenetic mechanisms in this age group are still little known.^1^^,^^8^^,^^10^

Smoking and alcohol drinking are well-established risk factors for head and neck cancer.^11^ Although this form of cancer affects mostly males, there has been a significant increase in the incidence in females, probably reflecting changes in smoking and drinking habits.^12^

The aim of this paper was to describe the social and demographic profile of head and neck cancer patients at a university hospital, and to identify the risk factors (smoking and alcohol drinking) to support disease prevention programs.

## SERIES AND METHOD

The Research Ethics Committee of our institution approved the research project (protocol 5566/2005).

A retrospective study was done of the medical files of head and neck cancer patients of the hospital Otorhinolaryngology Unit comprising six years between 2000 and 2005.

Variables were age, sex, occupation, skin color, smoking and alcohol drinking habits, primary tumor site, clinical staging, histological differentiation, treatment and patient mortality.

Tumors were classified according to the anatomical site in the mouth, the pharynx and the larynx. The oral cavity was divided into the lips, the anterior 2/3 of the tongue, the palate, the oral mucosa, the gingiva, the retromolar trigone and the hard palate. The pharynx was divided into the following three separate regions: the oropharynx (soft palate and uvula, the tonsils, and the lateral and posterior walls), the hypopharynx (piriform sinuses, hypopharyngeal wall, the postcricoid region and non-postcricoid areas), and the nasopharynx (lateral walls, choanae). The larynx is subdivided into the supraglottis, the glottis and the subglottis.^13^

Tumor staging (TNM) was done according to the guidelines of the American Joint Committee on Cancer (AJCC).14,15 Initially, the files of 427 patients were analyzed, but only 372 were TNM-staged.

Data were compiled in the software Microsoft Excel and analyzed by exploratory descriptive statistics.

## RESULTS

Data were collected from 427 patients seen between 2000 and 2005.

There were 367 male patients (86%) and 60 female patients (14%). Age ranged from 30 to 94 years (mean – 61.77 years; standard deviation – 11.44 years). Table 1 shows the age distribution of patients for each age group. Skin color was subdivided into two types (white and non-white). Non-white subjects were those with black, brown and oriental skin colors. In this study 90% of the subjects were white.

**Table 1:** Age distribution of head and neck cancer patients.

Age group	Number of patients (%)
30 a 40	9 (2,11)
41 a 50	66 (15,45)
51 a 60	121 (28,34)
61 a 70	127 (29,75)
71 a 80	82 (19,2)
81 a 90	20 (4,68)
> 90	2 (0,47)

The most frequent professional occupation in males was a rural activity (grower – 24.25%), followed by bricklayer (13.9%) and driver (11.17%). The most frequent occupation for females was housewife (60%) and rural activities (grower – 8.3%). Retirement with no specification as to the previous occupation was reported by 8.17% of males and 15% of females (Table 2, Table 3).

**Table 2:** Occupation of male head and neck cancer patients.

Occupation of males	Number of patients (%)
Grower	89 (24,25)
Bricklayer	51 (13,9)
Driver	41 (11,17)
Retired	30 (8,17)
Shopkeeper	16 (4,36)
Non-specialized services	13 (3,55)
Carpenter	11 (3)
Painter	9 (2,45)
Guard	9 (2,45)
Other	98 (26,7)

**Table 3:** Occupation of female head and neck cancer patients.

Occupation of females	Number of patients (%)
Housewife	36 (60)
Retired	9 (15)
Grower	5 (8,3)
Other	10 (16,7)

Smoking was a habit in 83.37% of the sample; 65.8% reported using alcoholic beverages; 55.27% of the sample reported having both habits; and 6.18% reported having neither habit. Quantities consumed were not documented.

Table 4 shows the primary tumor sites in 427 patients. The oral cavity had the highest rate, 35.37% (151 of the 427 cases).

**Table 4:** Primary anatomical site in head and neck cancer patients.

Anatomical site	Number of patients (%)
Oral cavity	151 (35,37)
Larynx	133 (31,15)
Oropharynx	69 (16,15)
Hypopharynx	36 (8,43)
Nasopharynx 8	8 (1,88)
Unknown primary site	30 (7,02)

Table 5, Table 6, Table 7 present tumor staging according to the malignant tumor classification (TNM) and their frequency in the primary tumor sites. This information was not reported in the files in 55 cases.

**Table 5:** Distribution of primary tumor anatomical sites according to the TNM classification (category T).

Category	Anatomical site	Number of patients (%)
	Oral cavity	26 (38)
	Larynx	26 (38)
T1	Oropharynx	9 (13)
	Hypopharynx	4 (6)
	Nasopharynx	3 (4)
	Oral cavity	39 (48)
	Larynx	27 (33)
T2	Oropharynx	11 (14)
	Hypopharynx	2 (2)
	Unknown primary site	2 (2)
	Oral cavity	32 (30)
	Larynx	38 (36)
T3	Oropharynx	17 (16)
	Hypopharynx	15 (14)
	Nasopharynx	2 (2)
	Unknown primary site	3 (3)
	Oral cavity	39 (41)
	Larynx	23 (24)
T4	Oropharynx	21 (22)
	Hypopharynx	10 (11)
	Nasopharynx	1 (1)
	Unknown primary site	1 (1)
	Larynx	2 (9,5)
Tx	Nasopharynx	1 (4,75)
	Unknown primary site	18 (85,75)

**Table 6:** Distribution of primary tumor anatomical sites according to the TNM classification (category N).

Category	Anatomical site	Number of patients (%)
	Oral cavity	94 (40)
	Larynx	87 (37)
N0	Oropharynx	31 (13)
	Hypopharynx	15 (6)
	Nasopharynx	4 (2)
	Unknown primary site	2 (1)
	Oral cavity	25 (42)
	Larynx	13 (22)
N1	Oropharynx	13 (22)
	Hypopharynx	6 (10)
	Unknown primary site	3 (5)
	Oral cavity	12 (23)
	Larynx	9 (17)
N2	Oropharynx	12 (23)
	Hypopharynx	7 (13)
	Nasopharynx	3 (6)
	Unknown primary site	9 (17)
	Oral cavity	5 (21)
	Larynx	6 (25)
N3	Oropharynx	2 (8)
	Hypopharynx	3 (13)
	Unknown primary site	8 (33)
Nx	Larynx	1 (33)
	Unknown primary site	2 (67)

**Table 7:** Distribution of primary tumor anatomical sites according to the TNM classification (category M).

Category	Anatomical site	Number of patients (%)
	Oral cavity	120 (36)
	Larynx	111 (33)
	Oropharynx	54 (16)
M0	Hypopharynx	23 (7)
	Nasopharynx	6 (2)
	Unknown primary site	19 (6)
	Oral cavity	1 (25)
M1	Larynx	1 (25)
	Unknown primary site	2 (50)
	Oral cavity	15 (43)
	Larynx	4 (11)
	Oropharynx	4 (11)
Mx	Hypopharynx	8 (23)
	Nasopharynx	1 (3)
	Unknown primary site	3 (9)

There was a predominance of squamous cell carcinomas (SCC), which was present in 96.7% of cases. Other histological types were also found, such as non-Hodgkin's lymphoma, undifferentiated carcinomas and others (Table 8).

**Table 8:** Histological differentiation of head and neck cancer patients.

Histological differentiation	Number of patients (%)
Squamous cell carcinoma (SCC)	413 (96,7)
Non-Hodgkin's lymphoma	3 (0,69)
Undifferentiated carcinoma	3 (0,69)
Other *	8 (1,92)

* adenocarcinoma, cystic adenoid carcinoma, clear cell carcinoma, mucoepithelioid carcinoma, poorly differentiated carcinoma.

Indications for radiotherapy or surgery generally balanced out for T1 and T2 tumors; most of the T3 and T4 tumors, however, required multimode treatment, usually surgery and adjuvant radiotherapy. On the other hand, other factors may have influenced somewhat the choice of treatment, such as age, professional voice users, uncontrolled smoking or alcohol drinking, as well as social and economic factors that might have required short-term solutions. At our unit treatment includes surgery, chemotherapy and radiotherapy. Most of the patients underwent surgery associated with radiotherapy (33.25%).

There were 164 deaths out of 427 cases; lack of information in the charts precluded a survey of the causes of death in most patients.

## DISCUSSION

Most of the subjects in our sample were aged between 51 and 70 years, which is similar to the data reported by the Head & Neck Unit of the Oncology Center, Oswaldo Cruz University Hospital, in the state of Pernambuco; their data shows that 55.82% of these tumors occur in this age group.^16^

Our finding that there is a higher incidence of head and neck tumors in males is similar to existing reports in the literature.^2^ Head and neck tumors are relatively rare in females,^17^ particularly in developing countries, where males predominate.^18^ In recent years there has been a significant increase in the incidence of head and neck tumors in females, probably due to changes in smoking and alcohol drinking habits.^12^ There were more rural workers among males, and more housewives among females. A survey undertaken at the Head & Neck Surgery Unit of the Heliopolis Hospital in the state of Sao Paulo revealed that the most frequent occupation among males was that of bricklayer, with rural activities in sixth place; the most frequent occupation for females was that of housewife, followed by rural activities.^19^ It should be noted that rural work exposes individuals constantly to the sun and to carcinogens, which helps promote cancer development.^19^^,^^20^ Another study done at the same hospital showed that about 85% of male and female patients were white.^19^ White was also the predominant skin color in our sample (90%).

Most of our patients were smokers (83.37%) and alcohol drinkers (65.8%), which strengthens the association between alcohol drinking and smoking and head and neck cancers.^1^ Many studies have shown a consistent relation between tobacco and alcohol, and cancer of the larynx and oral cavity.^1^^,^^21^, ^22^, ^23^

The most frequent tumor site in our series was the oral cavity (35.37%), followed by the larynx (31.15%). Epidemiological studies have also reported that 40% of head and neck cancers occur in in the oral cavity.^1^ This finding appears to reflect smoking and alcohol drinking habits, which may increase two or threefold the risk of these diseases in the oral cavity.^9^^,^^11^

The TNM classification of our series revealed that 25% of the cases were T3, 35.65% of the cases had lymph node involvement, and 2% presented metastases, showing that advanced disease was present upon the diagnosis. The literature reports that a high frequency of head and neck cancer cases are diagnosed at an advanced stage,24 as we ourselves also demonstrated. A Brazilian study, taking place in a developing country, shows statistically significant differences in these features compared to the findings of studies about patients from institutions in developed countries.^17^

The most representative histological type was the squamous cell carcinoma (96.7% of cases). Another study done in the state of Pernambuco has reported this type as the most frequent.^16^ The literature shows that over 90% of the cases of head and neck cancer are squamous cell carcinoma.^22^

Open surgery and external radiotherapy are fundamental approaches to treat carcinomas.^25^ Radiotherapy associated with surgery was the predominant approach in our series (33.25%), followed by radiotherapy only (28.10%). In another study, 528 out of 1,010 cases underwent surgery, 335 were treated by both surgery and radiotherapy, and 67 were treated by surgery, radiotherapy and chemotherapy.^25^ In our sample only 23 cases were treated with surgery associated with chemotherapy.

There were 164 deaths (38.4%) between 2000 and 2005. Head and neck cancer is characterized by local aggressiveness and by a high recurrence rate of secondary tumors with a high mortality rate.^10^

High-risk populations should be targeted for educational and surveillance programs. These programs and measures can attenuate the unfavorable outcomes in patients with mouth cancer, and decrease the risk of developing secondary tumors.

## CONCLUSION

The analysis of 427 patients seen at a university hospital between 2000 and 2005 is in agreement with data reported in the literature showing that head and neck cancer is more frequent in male smokers aged over 40 years. Medical data reveal that the primary site in most of the patients is the oral cavity. Identifying the risk factors for these patients may enable strategies for implementing prevention programs against this disease.
